# Calcium Biofortification of Crops–Challenges and Projected Benefits

**DOI:** 10.3389/fpls.2021.669053

**Published:** 2021-07-16

**Authors:** Marija Knez, James C. R. Stangoulis

**Affiliations:** ^1^College of Science and Engineering, Flinders University, Adelaide, SA, Australia; ^2^Centre of Research Excellence in Nutrition and Metabolism, National Institute for Medical Research, University of Belgrade, Belgrade, Serbia

**Keywords:** calcium, Ca biofortification, Ca deficiency, Ca in crop plants, Ca bioaccessibility

## Abstract

Despite Calcium (Ca) being an essential nutrient for humans, deficiency of Ca is becoming an ensuing public health problem worldwide. Breeding staple crops with higher Ca concentrations is a sustainable long-term strategy for alleviating Ca deficiency, and particular criteria for a successful breeding initiative need to be in place. This paper discusses current challenges and projected benefits of Ca-biofortified crops. The most important features of Ca nutrition in plants are presented along with explicit recommendations for additional exploration of this important issue. In order for Ca-biofortified crops to be successfully developed, tested, and effectively implemented in most vulnerable populations, further research is required.

## Calcium–An Essential Nutrient for Humans

Calcium (Ca) is the fifth most abundant inorganic element and accounts for about 2% of the total human body weight, and it is a vital nutrient for human health (Nordin, 1997). About 99% of Ca in the body is stored in the skeleton and the rest in the teeth and soft tissues, mostly as Ca phosphate (Nordin, 1997; Guo et al., 2014). The plasma level of total Ca is 2.2–2.6 mmol, while the amount of ionized Ca (i.e., Ca^2+^), which determines its biological potency, fluctuates between 1.1 and 1.4 mmol (Prasad and Shivay, 2020). Ca concentrations in plasma are regulated by vitamin D, calcitonin, and thyroid hormone (Prasad and Shivay, 2020).

Calcium is a cationic macronutrient and a structural component involved in a variety of biological and physiological processes in the human body. It plays a crucial role in the structure and signaling development of bones and teeth (Sharma et al., 2017). Some of the fundamental regulatory functions in the human body involve Ca, i.e., hormonal secretion, coagulation of blood, initiation of enzymatic reactions, vascular vasodilation, muscle function, nerve impulse transmissions, cell proliferation, and intracellular metabolism (Pravina et al., 2013). Ca is a critical element for the development of peak bone mass in adolescents and young adults and the retention of bone mass in older adults (Balk et al., 2017). Besides, Ca has a protective role against various types of cancers, i.e., colorectal, ovarian, breast cancer (Gao et al., 2005; Lin et al., 2007), and it reduces the risk of developing insulin resistance and cardiovascular diseases (Parikh and Yanovski, 2003).

Higher dietary Ca intake is associated with lower blood pressure, lower body weight, reduced adiposity, and a decreased risk of developing hypertension (McCarron, 1989; Parikh and Yanovski, 2003).

The recommended dietary intake of Ca is in the range of 800–1,300 mg/day for adults and 1,300 mg/day for children above 9 years of age [The Food and Agriculture Organization (FAO) United Nations, 2002].

Calcium requirements must be met *via* diet; nonetheless, dietary consumption of Ca in humans is very often lower than recommended (Kranz et al., 2007; Balk et al., 2017). According to the most recent systematic review of dietary Ca intake among adults across 74 countries, the average national intake of Ca was in the range of 175–1,233 mg/day (Balk et al., 2017). Ca intakes below 500 mg/day were reported for an adult population in Asia, while African and South American adults had Ca intake between 400 and 700 mg/day. Only people living in Northern European countries had Ca intakes above 1,000 mg/day (Balk et al., 2017). The majority of adolescent girls (90%) and boys (50%) in the United States have a suboptimal dietary intake of Ca, and the situation is even more alarming in developing countries (Vatanparast et al., 2010).

Calcium deficiency is most prevalent in low- and middle-income countries where access to Ca-rich foods is limited. Kenya, Bangladesh, South Africa, India, Indonesia, Vietnam, South Korea, and China are currently the most affected regions, with Ca intakes between 25 and 33% of recommended levels (Bromage et al., 2016; Dewey, 2016; Balk et al., 2017). More than 80% of the older adult population of South Korea has a Ca intake below the suggested values (Balk et al., 2017).

In 2011, Ca supply on a global scale was 684 ± 211 mg/capita/day with estimated 3.5 billion people (51 ± 32%) worldwide suffering from Ca deficiency due to insufficient dietary intake (Kumssa et al., 2015). People living in countries with lower purchasing power have been shown to have a higher Ca deficiency risk. Based on the global food supply data, the mean Ca deficiency risk was 80 ± 31 for Africa, 29 ± 27 for America, 57 ± 36 for Asia, 11 ± 7 for Europe, and 11 ± 4% for Oceania (Kumssa et al., 2015). Ca intake levels for over half of the countries worldwide are still unknown, which implies that the number of people affected by dietary Ca deficiency may be even larger than currently reported.

The consumption of staple foods with a limited Ca content and bioavailability is the major contributor to Ca insufficiency. Additionally, due to resource constraints, people living in developing communities, are not always able to pay for livestock or may only raise calves for complementing the income without consuming milk and milk products (Vatanparast et al., 2010). In developed countries where access to a variety of foods is not an issue, Ca deficiency mainly occurs as a consequence of avoidance and/or low intake of dairy products. Due to undesirable effects of lactose intolerance, a lot of people have limited consumption of the greatest dietary sources of Ca, milk, and dairy products. About 65% of the population of the world is lactose intolerant; hence, they cannot count on dairy products for their Ca requirements (Puranik, 2017). Consequently, a significant number of people, including vegetarians and vegans, require alternative food sources to meet their Ca needs.

Inadequate dietary intake of Ca in humans has been associated with various diseases, i.e., rickets in children and osteopenia and osteoporosis in adults (Chan et al., 2007; Pettifor et al., 2018). Poor intake of Ca and Ca deficiency has numerous health and economic consequences (Kranz et al., 2007). The World Health Organization (WHO) has acknowledged osteoporosis as the ensuing public healthcare concern globally, affecting nearly 75 million people in Europe, the United States of America, and Japan (Haldipur, 2003). The worldwide cost of handling osteoporosis is estimated to be $131.5 billion USD by 2050 (Lindsay et al., 2001).

Calcium deficiency in humans can be treated by increasing dietary Ca intake and absorption. Diversification of diets, food fortification, supplementation, and crop biofortification are strategies that could help alleviate Ca malnutrition (Puranik et al., 2017). While effective, both food fortification and supplementation have some drawbacks, the major being the inability to be easily accepted and applied by people in developing countries, and require ongoing infrastructure and investment. Alternatively, more cost-effective solutions with long-term benefits should be pursued.

In general, 50–70% of dietary Ca supply is from animal products, with fruits and vegetables contributing 10–40% of dietary Ca intake while cereals provide very little Ca (Welch and Graham, 1999; Khush, 2001). In order for people to absorb the recommended daily amounts of Ca, they need to consume Ca-rich foods with easily absorbable types of Ca. Despite being the richest sources of dietary Ca, the availability and the consumption of milk and dairy products are inadequate for different reasons, i.e., lactose intolerance or limited access to dairy foods. In addition, absorption of the available Ca from milk and dairy products is often limited because of the Ca–Mg competition for absorption (Bennett and Sammartano, 2005). Plant-based foods provide Ca that is much more easily absorbed (Lanham-New, 2006).

Half of the population of the world depends on cereals as a basic food source (FAO et al., 2020). Fifteen crop plants are covering 90% of the total food energy intake worldwide. Major food crops are important to millions of people in developing countries, with three of them (i.e., rice, maize, and wheat) providing 60% of the food energy intake of the world (FAO et al., 2020). Staple crop self-sufficiency becomes a central element of national agricultural policies (FAO et al., 2020); thus, the production and application of Ca-biofortified staple crops could be a potential long-standing solution for alleviation of the Ca malnutrition problem worldwide.

## Calcium in Edible Crops

Plant-based products are the largest potential sources of more readily available and absorbable forms of Ca (Weaver et al., 1999; Lanham-New, 2006). The concentration of Ca in plant-based foods shows an extensive range of variation (Lanham-New, 2006). Two conditions must be met to classify a food as a good source of Ca. A typical serving size must comprise of at least 30 mg of absorbable Ca, and 100 kcal of food must deliver 30 mg of absorbable Ca (Fleming and Heimbach, 1994). In addition, diets should provide 200 mg/100 g of Ca to neutralize the negative impact of phytic acid (McCance and Widdowson, 1942). Processed flour should contain 235–390 mg/100 g of Ca, with an exclusion of wholemeal flour where the Ca content of 200 mg/100 g is considered acceptable (Broadley and White, 2010).

The lowest Ca content is found in fruit (i.e., apples and tomatoes) and tubers (potatoes), averaging 10 mg of Ca per 100 g. Much higher values are seen in green leafy vegetables (i.e., endive and spinach), with approximately 78 mg/100 g (Martínez-Ballesta et al., 2010). Similarly, bok choy and broccoli are good sources of Ca (Yang et al., 2012). Certain foods like ivy gourd (*Coccinia grandis*), kale (*Brassica oleracea*), and Chinese mustard greens (*Brassica juncea*) have comparable Ca content and absorbability to dairy products (Dayod et al., 2010).

Cereals are major food sources for people in developing countries, and, as such, the largest potential sources of easily available Ca. Similarly, cereals and cereal-based products contribute substantially to the dietary intake of total Ca in developed country populations. Around 37% of the total Ca intake of men and women living in Greece was coming from cereals (Welch et al., 2009).

Likewise, 28% of dietary Ca intake of people in United Kingdom was drawn from cereals and cereal-based products; 28% of the dietary intake of Ca in females and 32% of the Ca intake of males are based on cereal sources (Broadley and White, 2010). Therefore, potential increases of Ca bioaccessibility in cereal crops could provide notable benefits, both to developing and developed country populations.

**FIGURE 1:**
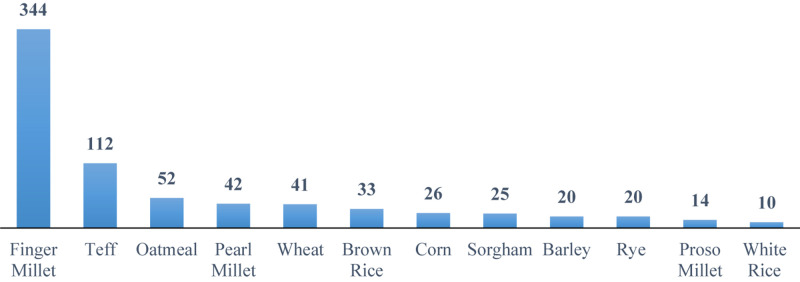
Ca content of different crops (mg/100 g edible portion). Based on data provided by McKevith (2004), Saleh et al. (2013), and Shobana et al. (2013).

Major staple food grains (i.e., rice, wheat, and maize) that make up the central part of diets within both developing and industrialized countries are relatively poor sources of Ca (Jeong and Guerinot, 2008). Ca content of major crop plants is presented in Figure 1. The exception to the rule of relatively low Ca content of cereals is finger millet (Eleusine coracana), a crop cultivated in Eastern and Central Africa and India that contains three times more Ca than milk, 344 mg/100 g (Seetharam, 2001). Ca concentration in 36 genotypes of finger millet varied from 162 to 489 mg/100 g with a mean value of 320 mg/100 g grain (Upadhyaya et al., 2011; Sharma et al., 2017). White-seeded finger millet had much higher Ca concentration when compared with the brown-seeded varieties; 330 mg/100 g as opposed to 296 mg/100-g grain (Seetharam, 2001). As such, finger millet has enormous potential as a nutritional security crop due to the unusually high Ca concentration (Sharma et al., 2017). Furthermore, millets are often resistant to pests and diseases, and are drought-tolerant crops, however, the major downside of finger millet is the predominance of antinutritional factors that reduce Ca bioavailability (Vinoth and Ravindhran, 2017). Besides being a great source of Ca, this crop has the potential to be used as a model to examine the mechanisms that contribute to high Ca concentration in grains.

The green revolution-stimulated research on staple foods and the lower-yielding cereals and legumes have been replaced with higher-yielding varieties that are generally poorer sources of essential nutrients, including Ca (Graham et al., 2007). The consumption of 100 g of a cereal (i.e., wheat) provides an intake of 40–50 mg of Ca, which is equivalent to 5–6% of the recommended daily requirements (Sanwalka et al., 2011). One processed serving portion of finger millet provides 0.2-g Ca, which is 25% of the recommended intake of Ca for children and adolescents (Sanwalka et al., 2011; Ekbote et al., 2017).

In summary, staple crops that could provide sufficient amounts of Ca, predominantly to people of low-income groups, are required. These populations are usually dependent on the foods they produce for meeting their Ca needs, so Ca-rich, traditional, and locally well-adapted crops are desired. Regular consumption of finger millet has a great potential to control the occurrence of Ca deficiency. Similarly, the action toward increasing the potential of other major crops to become promising food sources of Ca should be stimulated. Finally, the development and application of Ca-biofortified staple crops should be considered as an approach to improving dietary Ca intake of the most vulnerable populations.

## Calcium in Plants

Comprehensive explanations of the role of Ca in plants, Ca transport mechanisms, Ca transporters in cellular membranes, Ca channels and signaling, Ca binding proteins, and phylogeny of shoot Ca concentrations within the plant tissues are offered elsewhere (White, 2001; White and Broadley, 2003, 2005; Mohiyuddin et al., 2010); therefore, only the most important aspects are covered here very briefly.

Plants require relatively large amounts of Ca, typically from 0.1 to 4.4% dry matter (Broadley et al., 2003). Still, Ca deficiency in plants/field-grown crops is generally rare, but it could be seen in the plants grown in the soils with high levels of acidic depositions or low-based saturation and in the leaching prone soils (McLaughlin and Wimmer, 1999). Ca deficiencies can occur on highly exposed tropical soils, on sodic and saline soils, when Ca is reduced in the presence of other cations, i.e., Na or Al (Broadley and White, 2010). Deficiency of Ca in crops also arises when the Ca is temporarily inaccessible to the growing tissues (Ho and White, 2005). Plants can suffer from Ca toxicity if excessive amounts of Ca are present in a rhizosphere solution; the abundance of Ca oxalate crystals in the cell walls increases with high Ca fertilization and under conditions of high humidity (White and Broadley, 2003). Most of the Ca-based components found in the soils are insoluble and consequently unavailable to plants, as Ca is taken from the soil solution in the form of Ca^2+^.

The mass flow of water transports Ca toward the root and once in the root/the cell wall space, Ca^2+^ either binds to negatively charged residues within the Donnan-free space or to membranes. Furthermore, Ca is transported across the cell plasma membrane down the electrochemical gradient for Ca^2+^ and is simplistically delivered to the xylem (White and Broadley, 2003). Ca is transported *via* the apoplastic pathway to the xylem (White, 2001), while a symplastic pathway permits the cells to control the rate of Ca transport to the shoot by the plasma membrane Ca^2+^-ATPases or Ca^2+^/H^+^ antiporters of the cells within the stele (White, 2001). Ca is immobile in the phloem, so tissues that do not transpire easily (i.e., fruits, seeds, and tubers) have low Ca concentrations due to low transpiration rates in these tissues (White and Broadley, 2003; Karley and White, 2009).

Once in the plant cells, Ca is relatively immobile and is not easily circulated to growing parts of the plants, which can lead to local deficiencies, despite the adequate supply of Ca. This causes many side effects, local plant cell necrosis, reduced plant ability to cope with abiotic and biotic stress, decreased crop quality, and reduced yield (White and Broadley, 2003; Dayod et al., 2010).

Calcium is differentially distributed within different cell types but also intracellularly, with organelles, i.e., vacuoles, mitochondrion and an endoplasmic reticulum, having a higher Ca concentration than the cytosol (Dayod et al., 2010). The exchange capacity of the cell wall for Ca depends on the tissue type and environmental conditions (Fritz, 2007); however, the soluble Ca is generally located in specific cells of the shoot (Karley et al., 2000). For example, in cereals, total Ca is lowest in the mesophyll and highest within the epidermal leaf vacuoles (Karley et al., 2000).

Several techniques have been suggested for increasing Ca uptake by plants. One of the ways to accomplish this is by increasing the mass flow of water and Ca to the roots, a process often limited by a low supply of water in developing countries (White, 2001; Puranik et al., 2017). An alternative method is to increase the binding of Ca to cell walls or improve the entry of Ca into the cell walls by increasing the activity of Ca-permeable transporters (White, 2001). In this context, increasing the cation-exchange capacity (CEC) of the cell walls is a strategy that could bring more Ca into plants (Fritz, 2007). CEC of cereals is low, which could explain their low-Ca content. Ca concentrations in plant shoots increase several folds in plants exposed to Ca-rich habitats without negative effects on plant growth (Jefferies and Willis, 1964). While this sounds like a promising strategy, the undesirable effects of this approach are still not known as Ca-signaling pathways are not entirely understood, and the proposed overstimulation could disturb tightly regulated transport of Ca within plant cells (Dodd et al., 2010; Yang et al., 2012). The Ca accumulation in plants is regulated by active tonoplast processes (Conn and Gilliham, 2010).

The largest potential storage compartment in the plant is the vacuolar lumen, so it is usually chosen for increasing plant Ca storage capacity, with CA (Ca-ATPase) and ACA (autoinhibitory Ca ATPase) being the most important proteins for Ca accumulation into the vacuoles (Leigh and Sanders, 1997; Conn and Gilliham, 2010).

There are several approaches to increasing the Ca concentration and bioavailability of staple food crops, and they include the following: increasing Ca supply to cells; improving the Ca uptake by cells; removing the antinutrients (compounds that make Ca inaccessible); and increasing Ca storage at the cellular and tissue level (Yang et al., 2012; Puranik et al., 2017).

The presented findings indicate that strategies for improving Ca uptake and storage of crop plants exist, but most of these mechanisms are not completely developed and require further research.

All available methods for increasing Ca uptake and storage of plants require careful consideration of potential side effects.

## The Interaction of Calcium With Other Nutrients

The interaction of Ca with other nutrients and potential antagonistic or synergistic effects of their interplay are currently poorly understood. The ability of Ca to bind phytate and consequently improve the absorption of other major minerals, i.e., Zn, Fe, or vice versa is still inconclusive.

Multiple nutrient germplasm data have recently been evaluated for pearl millet, demonstrating that out of the 10 high-Fe accessions, only one accession had more than 58 mg/kg Zn, and four accessions were with higher-Ca content, 201–235 mg/kg (the lowest content measured was 161 mg/kg) (Govindaraj et al., 2020). Ca content of pearl millet grains had a positive association with concentrations of Fe, Zn, and many other examined nutrients, but significant associations were only evident for Fe and Mn (Govindaraj et al., 2020). Lentil seed Ca concentrations are directly correlated with Mg and Zn concentrations but negatively with Fe (Toklu et al., 2017). Strong positive correlations were seen for Ca in Arabidopsis thaliana, where Ca correlated with Mg, Mn, P, and Fe (Vreugdenhil et al., 2004). An examination of the element-binding affinities of numerous elements to phytate at pH 7 demonstrates that Ca-binding constants are much lower compared with binding capacities of other elements (Cu > Zn > Mn > Fe > Ca), signifying that a better release of Ca through the gastrointestinal tract can occur (Torres et al., 2005).

Calcium has a significant protecting role against Cd toxicity in plants (Huang et al., 2017) as Ca controls Cd uptake and translocation in a plant species in a dose-dependent manner (Gong et al., 2016). Lower doses of Ca promote Cd uptake and translocation in plants, but higher doses reduce the Cd uptake in the roots (Eller and Brix, 2015). The accumulation of Cd in plants was significantly reduced with increasing Ca concentrations in rice plants; Ca diminished root Cd uptake and stimulated translocation of Cd from roots to shoots (Zhang et al., 2020a).

The potential impact of modifications in Ca intake on dietary Fe bioavailability showed that both heam and non-heam Fe absorption were inhibited by dairy products and Ca supplements (Hallberg et al., 1992; Thompson et al., 2010). The inhibitory effect of Ca on Fe absorption is most pronounced in small, simple meals but is less noticeable when larger complex meals are consumed, most likely because of the combined effect of all modifiers of Fe absorption in a composite meal (Fairweather-Tait and Hurrell, 2007). However, Ca supplementation has a negative effect on Fe absorption only if the habitual Ca consumption is very low; otherwise, the influence is not significant (Lynch, 2000).

The inconsistent findings on the influence of higher Ca concentrations on the absorption of major micronutrients shows that complex nutrient interactions exist, suggesting further research is needed to clarify the effect of Ca on the availability and absorption of other important toxic and non-toxic elements. A better understanding of the mechanistic interactions of a number of microminerals within the crop plants tissues is needed. The optimal conditions for stimulating the desired interactions, i.e., soil properties, climate, Ca concentrations, need to be determined to produce Ca-biofortified crop varieties with the most desirable traits. The micronutrient interactions at the human gut level should also be taken into account.

## Antinutrients and Bioavailability

Calcium bioavailability depends on both the food source and the presence of anti-nutritional factors. The presence of anti-nutrients can bind elemental nutrients and prevent absorption in the gut. In plants, Ca is complexed with phytate, fiber, polyphenols, proteins, fatty acid, lactate, and oxalate (Krishnan et al., 2012; Yang et al., 2012). The formation of Ca-oxalate crystals makes Ca completely inaccessible for uptake and absorption along the length of the gut, so oxalate is referred to as a strong “antinutrient” (Heaney and Weaver, 1989). Plant species deposit Ca-oxalate crystals in their vacuoles or they hold soluble oxalate in the form of sodium and potassium salts. Due to the complexation of Ca with oxalate, Ca is indigestible from the many edible plants high in Ca (Morris et al., 2007).

Phytic acid, known as “phytate” or “inositol hexakisphosphate” (IP6) is another major antinutrient of Ca in plants. Phytate chelates Ca and makes complexes that humans cannot digest. High concentrations of phytate in plants can cause Ca deficiency and malnourishment, even if the concentration of Ca within the edible part of the plant is adequate. Unrefined cereals and legumes contain the highest concentrations of phytate (600 mg/100 g of dry weight) (Lott et al., 2009). Dephytinisation can enhance the amount of absorbable Ca in plants, but not to a point to overcome the deficits of low-Ca content of plant-based foods used in developing countries (Gibson et al., 2010).

Phytate to Ca molar ratios are used to predict Ca bioavailability; the phytate:Camolar ratio above 0.24 is shown to impair Ca absorption (Morris and Ellis, 1989). Food processing approaches, such as germination, fermentation, and thermal treatments, are generally seen as promising tools for increasing Ca bioavailability, as they tend to reduce the amounts of phytate and improve Ca bioaccessibility (Zhang et al., 2020b).

Phytase, an enzyme that catalyzes the hydrolysis of phytic acid into inositol phosphate intermediates, could be present within germination seeds or formed throughout the microbial activity, and thus phytases are the main determining factors of Ca bioavailability (Zhang et al., 2020c). Ca bound to indigestible food components could pass to the colon and might turn out to be absorbable through enzymatic reactions of microbiota (Weaver et al., 1999). For example, probiotics, containing *bifidobacterium, lactobacillus*, and *bacteroids* increased Ca balance in the gut and contributed to improvements in bone mineral content (Scholz-Ahrens, 2016). The Ca content in the bones of animals improved when *lactobacillus paracasei*. *L. plantarum* or *bifidobacterium longum* was supplied to rats (Parvaneh et al., 2015). Probiotics increase absorption of Ca by producing SCFAs that improve Ca release and absorption (Asemi and Esmaillzadeh, 2013). SCFAs, such as acetate, butyrate, and propionate, enhance Ca solubility and increase the expression of Ca transporters (Skibsted, 2016). The bioavailability of Ca in certain plants could be higher than anticipated, and it requires to be adequately assessed. For example, Ca in Chinese cabbage is found to be 1.3 times higher bioavailability than milk Ca (Kamchan et al., 2004).

Phytase treatment of beans improved the amount of absorbable Ca by a third (Weaver et al., 1993). Reduction of phytic acid in soybean seeds of 70% increased Ca bioaccessibility by around 33% (Heaney et al., 1991). High Ca bioavailability was measured in Brassica vegetables that are known as phytate and oxalate free vegetables (Lucarini et al., 1999). Chickpea genotypes with lower levels of phytic acid and phenolics had increased Ca bioavailability, regardless of their Ca concentrations in the seeds (Sharma et al., 1996). Chickpea is a seed with a relatively low phytate content and a good source of mineral elements, including Ca, so this plant could be a favorable candidate for increased concentrations of Ca in breeding programs (Dragicevic et al., 2018). However, the concentration of phytate alone is not always responsible for inhibited Ca bioavailability, while a synergistic role of protein and phytate seems to have a fundamental role in the context of Ca bioavailability. Similarly, the Ca:phytate molar ratio is not the best predictor of Ca bioavailability, as phytate is modified by other food ligands, proteins, and other interacting cations (Zhang et al., 2020b).

Low-phytic acid (lpa) crops could genetically be produced, but phytic acid contains certain health-promoting properties for both plants and humans, so simply removing the phytate may not provide the desired long-term outcomes (Bowen et al., 2007). Phytates play a major role in plant metabolism, growth, and resistance to biotic and abiotic stresses (Singh et al., 2017). Phytic acid is the main phosphorous store for the seeds and is often linked to adverse effects on the plant (Singh and Raghuvanshi, 2012). In humans, phytate has a protective role against various chronic diseases (Kumar et al., 2016).

However, it is important to note that certain perturbations in phytic acid synthesis may beneficially affect the distribution of minerals within the cereal grain tissues and result in higher mineral levels in the endosperm (Cichy and Raboy, 2009). Dietary phytate certainly has beneficial roles in human health but may not always be to the degree as it is often claimed (Raboy, 2020). Besides, the benefits of low phytate foods should not be ignored and neglected (Raboy, 2020), and breeding for elevated levels of Ca may not be effective if phytic acid levels remain high (Raboy, 2020). For example, phytate levels in the various grain seeds are not well correlated with Fe and Zn levels; however, animal and human studies describe that low-phytate grains may result in enhanced absorption of minerals (Raboy, 2020). A combination of low phytate with a high mineral approach may provide a more suitable solution for improving the long-term global bioavailability of Ca in staple crops and for improving Ca nutrition of most vulnerable population groups, an idea that requires further consideration.

The previously discussed strategies have the potential to increase the content, accumulation, and bioavailability of Ca in target crops, but most of these procedures are expensive and they can hardly be applicable to be applied by developing countries. The biofortification approaches that enhance Ca concentration and Ca bioavailability of staple crops are more likely to be adopted in low-income societies and to bring the desired outcomes. Successful biofortification of crops with Ca requires a comprehensive understanding of the physiological and genetic basis of Ca accumulation in staple foods. Further work is needed in order to produce crop plants with higher Ca content without negatively affecting plant functioning and yield. Techniques that regulate Ca transport and storage in plants are still not entirely understood, so further research of potential mechanisms that control the transport and storage of Ca in plants is necessary. The role of molecular breeding, genomics, and transgenic approaches should be explored to understand the mechanisms of Ca accumulation in selected staples. Identification of potential candidate genes and controlling elements needed for elevated Ca accumulation in the grains is required. Genetic engineering and various imaging approaches could be used for studying the distribution of Ca and changes in the chemical forms of Ca in the plant cells.

The manipulation of the expression and activity of particular Ca transporters within the plants could lead to increased supply, uptake, and accumulation of Ca within crop plants, but this requires additional investigation. The reduction of antinutrients during plant growth and development is a strategy for increasing bioavailability from Ca-rich crops. The production of low phytate and high Ca staples may provide the best suitable solution; however, their application should be approached with caution to avoid the potentially negative effects of low phytate on the health status of both plants and humans.

## The Effect of Processing and Cooking Procedures on the Calcium Content

The food preparation and processing techniques affect the total Ca content and Ca bioavailability of crop grains. Decortication (removal of the seed coat matter) of finger millet lowered Ca content but increased the bioaccessibility of Ca by 15 g/100 g (Krishnan et al., 2012). The seed coat of finger millet grains contains 40 g/100 g of total Ca, so its removal reduces total Ca content to a degree (Krishnan et al., 2012).

Cereal grains are naturally low in Ca concentration, with the husk and brain, containing the highest concentrations of Ca, very often removed during milling procedures (Broadley and White, 2010). However, parboiling before milling may be a useful strategy for increasing the Ca content in the endosperm, as, during the process, Ca from its natural place of residence in the bran and aleurone layer moves to the endosperm (Oli et al., 2016). Autoclaving causes a considerable reduction of Ca concentration in lentils (Hefnawy, 2011).

Sonication has been shown to notably increase the Ca content of apple juice (Abid et al., 2014). No significant differences in Ca content were seen between the fresh and frozen products while certain reductions in Ca levels were observed during prolonged storage periods of fruits and vegetables, most likely caused by a moisture loss (Bouzari et al., 2015).

Milling and conversion of grains into other food products cause hydrolysis of IP6 to lower inositol phosphates (IP5-IP1) and produce products with enhanced bioaccessibility of Ca. Short-term high-temperature treatment of finger millet grains had no negative effect on Ca content but lowered Ca bioaccessibility by 19% (Krishnan et al., 2012). Similarly, microwave cooking by boiling did not improve Ca bioavailability (Amalraj and Pius, 2015). Popping the native millet for the production of a product named “Hurihittu,” slightly decreased the bioaccessibility of Ca by 7 g/100 g without affecting the phytic acid content (Krishnan et al., 2012).

Malting of millet decreased the total Ca content by 20 g/100 g, reduced the content of antinutrients (phytate, 84 g/100 g; dietary fiber, 81 g/100 g) and consequently significantly improved the bioaccessibility of Ca, 68 g/100 g (Krishnan et al., 2012).

Similar to malting, the germination process lowered Ca content, reduced phytic acid, and increased the bioaccessibility of Ca (Platel et al., 2010; Krishnan et al., 2012). Germination reduced the Ca:phytate and Ca:oxalate molar ratios in flaxseeds and increased bioaccessibility of Ca was observed with an *in vitro* gastrointestinal digestion process; however, there were no increases in Ca absorption in the animals (Vinco Pimenta et al., 2020).

Sprouting has been shown to reduce the phytate levels and improve the extractability of Ca (Mbithi-Mwikya et al., 2000). Whole grain finger millet flour had a much higher Ca content than the flour made from decorticated grains (Hemanalini et al., 1980). Furthermore, decortication improved Ca bioavailability (Hemanalini et al., 1980). In addition, the fermentation process improved Ca bioavailability by 20% and reduced the inhibitory activity of antinutrients, phytates primarily (Makokha et al., 2002).

Out of all processing procedures, milling, malting, and germination were the most efficient techniques for improving Ca bioaccessibility of finger millet, and this was primarily due to reduced concentration of antinutrients as a consequence of applied procedures. An increased phytate:Ca molar ratio decreased the bioaccessibility of Ca, even when the molar ratio was below the estimated critical value of 0.24 (Krishnan et al., 2012; Ma et al., 2016).

Calcium fortification processes started to be employed in the Latin America region as early as 1,200–1,500 BC by the so-called “nixtamalization” process (Naila et al., 2019). Nixtamalization, the procedure of soaking and cooking cereals in a lime solution, has been shown to increase Ca content of grains and to improve Ca absorption (Arnaud et al., 2007; Naila et al., 2019). Naila et al. (2019) have recently demonstrated that the Ca content of rice can be improved by fortification of rice with different concentrations of quenched lime. Cooking rice grains in a lime solution significantly improved the Ca content (Naila et al., 2019). Similar findings were provided for corn grains when they were soaked and cooked in a lime solution before dehulling (Bressani, 1990). The Ca content of tortillas made from nixtamalized corn flour was increased by 22–38-fold compared with the Ca content of tortillas made from conventional corn flour (Rosado et al., 2005).

In addition, the absorption of Ca was enhanced (Rosado et al., 2005), which was most likely stimulated by reduced concentrations of antinutrients (i.e., phytate, oxalate, and dietary fiber) in soaked grains (Rosado et al., 2005; Naila et al., 2019).

Calcium carbonate and Ca chloride have also been used as nixtamalization agents, and experiments conducted on maize plants showed that treatment with these agents could lead to modifications in the digestibility of resistant and soluble starches (Roldan-Cruz et al., 2020). Cooking reduces the oxalate content of the food by discharging losses into the cooking water (Savage et al., 2000). Concentrations of Ca significantly decreased upon cooking sweet chestnuts (Gonçalves et al., 2012).

The soaking time was positively associated with the Ca content of corn grains (Palacios-Fonseca et al., 2009) and millet flour (Ocheme et al., 2010), while no such association was found for rice plants (Naila et al., 2019). Soaked grains produced flour with a lower content of proteins, phytic acid, trypsin, and tannin inhibitors (Ocheme et al., 2010), showing that soaking could diminish some antinutritional properties. Organoleptic properties of Ca-modified lettuce varieties were not changed, so this suggests that biofortification of crop plants with Ca does not affect taste and flavor (Morris et al., 2008).

A careful selection of appropriate processing methods is required to preserve the maximum bioaccessible amounts of total Ca in designated cereal grain products. Further research is needed to extrapolate the procedures with the most beneficial effects on Ca content and bioaccessibility and with a minimum negative impact of reduced antinutrients content both for plant development and human consumption. Processing methods with the most substantial positive outcomes on Ca bioaccessibility should be selected and promoted among consumers.

## Environmental Effects on Calcium in Target Crops

The influence of environmental conditions on the Ca content of cereal plants has not been investigated extensively. The limited available evidence points out that the highest concentrations of Ca in the grains of wheat plant seeds were obtained during the most humid year. The concentration of Ca in wheat grains varied by 40% between the dry- and humid-seasons-grown wheat grains (Gomez-Coronado et al., 2019). The effect of crop season on the Ca content of common beans showed that the mean Ca content in the dry crop season was 2.6 times higher compared with rainy crop-season-grown counterparts (Fernandes et al., 2016).

Climatic conditions are known to affect the concentrations of phytate in plants. Wider variability in phytate content is due to climatic rather than genotypic variations. However, inconsistent data are obtained; the lowest phytate levels were measured in the most humid year by some (Gomez-Coronado et al., 2019), while others have found the lowest concentrations in the driest year (Ficco et al., 2009). The yield was increased during the humid years, and a phytate:Ca ratio showed the highest values during the driest season (Gomez-Coronado et al., 2019). The relationship between the Ca content of crops and soil characteristics is affected by soil Ca content, mineral bioavailability, and plant function (Wood et al., 2018). Crop Ca concentration was directly associated with soil pH, with higher mineral availability in plants grown on acidic soils (Fordyce, 2005). An increase in soil pH improves the relationship between the extractable soil Ca and crop Ca concentrations (Bevis and Hestrin, 2020).

The effect of root interaction with a particular soil type is another environmental factor that may influence the Ca sensing and transport mechanisms in a different way. Crops grown on calcareous soils had higher Ca content than the crops grown on non-calcareous soils (Joy et al., 2015). However, the characteristics of the soils do not seem to be the main drivers of heterogeneity in crop Ca concentrations. The relationship between the soil characteristics and the crop Ca concentrations mainly depends on the crop variety, climate, and agricultural practices, but additional research is needed to fully support this preliminary idea (Bevis and Hestrin, 2020).

Certain genotypes of crop plants have the potential to accumulate Ca under specific environmental conditions, and these genotypes should be used in breeding programs to produce biofortified grains and to help alleviate Ca deficiency in affected populations. The effect of various agronomic (vegetative growth, disease resistance, and stress resilience) and ecological (climate change) conditions on the Ca content of crops should be additionally examined. Mapping environmentally variable traits in a genetically highly diverse array of crop plants remains a challenge.

## Analysis of Calcium in Enriched Crops

Calcium has multiple roles in plant cells; thus localization, abundance, and speciation vary among the Ca complexes involved in different processes. Furthermore, it is thought that Ca-enhanced foods have altered Ca distribution and speciation in the tissues.

Calcium distribution in edible crop plants is usually assessed, using inductively coupled plasma-optical emission spectrometry (ICP-OES), inductively coupled plasma-mass spectrometry (ICP-MS), atomic absorption spectrometry (AAS), synchrotron X-ray fluorescence (SXRF) or X-ray absorption spectroscopy (XAS), that display all of the forms of Ca, regardless of their solubility (Yang et al., 2012). SXRF is particularly suitable for analysis of the membrane transport proteins, and it allows measurement of Ca in fresh, fully hydrated, or living tissue (Yang et al., 2012).

X-ray fluorescence (XRF) is shown to be a reasonably high-throughput method for measuring Ca in plant tissues (Kalcsits, 2016). The correlation coefficient ranged from 0.73 to 0.97 in apple and pear fruit, between Ca measurements taken by XRF and Ca values determined *via* traditional laboratory analysis (Kalcsits, 2016). XRF can be employed as a rapid easily applicable analytical technology for the determination of minerals, including Ca (McCarthy et al., 2020). Similarly, XRF technology can be used to explore the efficacy of biofortification methods in improving Ca concentrations and distribution within the grains, and it could be employed for analyzing the interaction of Ca with antinutrients and for examining interactions among multiple elements (Zhang et al., 2017; Vigani et al., 2018).

X-ray fluorescence is appropriate for examining the concentration and distribution of various elements within plant tissues (Guild et al., 2017; Feng et al., 2020). It allows for quick analysis of elements in the vegetation stages of plants. Handheld XRF is a portable device that allows a rapid assessment of Ca concentrations and variability on the surface and special distribution of Ca at the organ or plant level (Feng et al., 2020).

The benefits of using XRF techniques for the determination of Ca concentrations in crops are the following: it is a fast, high throughput method that can measure several elements in parallel, minimal sample preparation needed, no chemical reagents are needed, samples are not damaged, and there is no waste produced; the procedure does not change the chemical speciation and distribution of elements (Feng et al., 2020). Synchrotron XRF offers good sensitivity and excellent special resolution. Some downsides are that the long-term exposure to X-rays may damage the sample, and, sometimes, superimposed peaks occur due to interference with other elements (Feng et al., 2020).

The position of the grain affects the concentration of Ca within a crop plant, and this could have some important implications for plant breeding strategies. Ca concentration decreases as the distance from the rachis increases. The lowest Ca concentrations were found in distal grains that mainly contribute to grain yield while the wheat grains positioned more distally from the rachis contained 30% lower concentrations of Ca (Calderini and Ortiz-Monasterio, 2003). The concentration of Ca declines through grain filling, so, in wheat plants, Ca concentrations on the first sampling date were 70% of their value at physiological maturity (Calderini and Ortiz-Monasterio, 2003). Higher concentrations of Ca are found in the bran and germ in raw rice while the endosperm has a low Ca content (Oli et al., 2016).

Understanding the link between the location of Ca in the plant cell and Ca bioavailability is important to be known so that molecular targets controlling Ca position in plants can be developed.

## Enrichment of Staple Food Crops With Calcium

Intervention programs that include nutrition education, fortification, and supplementation have been effective in alleviating various micronutrient deficiencies; however, they are expensive, entail persistent support, and very often fail to reach all individuals at risk (Graham et al., 2007). Several economic, political, and logistical challenges tend to be present and discourage the appropriate implementation of these strategies (Graham and Welch, 1995). The low-income countries lack robust food-processing infrastructure, and fortified products are usually unaffordable to the poor (Velu et al., 2014). In addition, the regulatory systems that could support the implementation of fortified foods in developing countries are frequently lacking (Graham and Welch, 1995). Ca supplements, while efficacious, will only be of use to informed, well-motivated, and educated consumers, and, as in every other form of supplementation, are less likely to be beneficial in disadvantaged areas (Yang et al., 2012).

**FIGURE 2:**
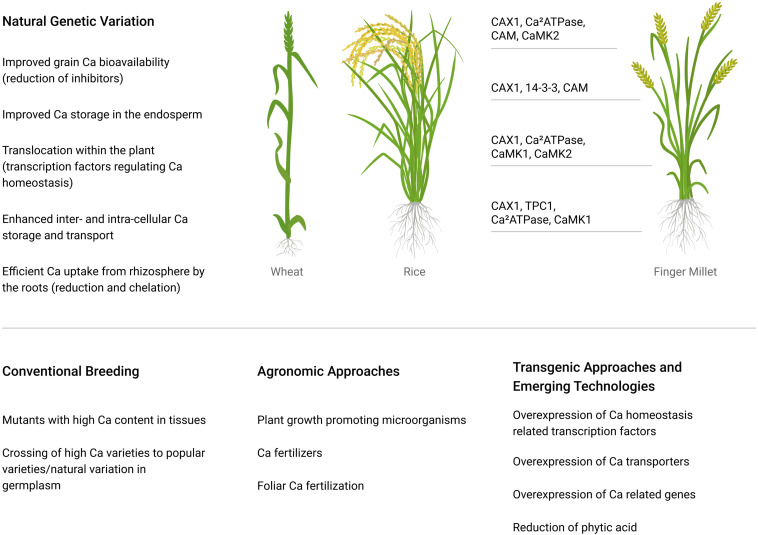
Potential mechanisms to improve Ca content of major crops. The transporters in finger millet have been studied more extensively, and they are presented lining up to the regions of the plant to where they appear to be expressed; data taken from Mirza et al. (2014) and Puranik et al. (2017).

Wheat and rice are the two most commonly consumed cereal crops worldwide (FAOSTAT, 2017). Around 765 million tons of wheat and 509 million tons of rice are produced per year (FAOSTAT, 2017). Rice is the main staple food for half of the population of the world (Juliano, 1990). Populations dependent on rice get 75% of their daily dietary energy through its consumption (FAOSTAT, 2017). Consequently, increasing Ca in edible grains has the potential to impact a large number of people. This can be achieved by several means, including fortification (adding a nutrient to the grain), biofortification (utilizing convention breeding approaches to increase the amount of a nutrient the plant produces in the seed), various genomic and transgenic approaches (Figure 2).

As shown by Broadley and White (2010), 50% increase in Ca content of rice is achievable and could bring Ca intake above the recommended values for 0.3 million people (Broadley and White, 2010). Ca fortification of rice is a potential solution for alleviation of Ca insufficiency in sensitive populations. Recently, Naila et al. (2019) have demonstrated that consumption of 300 g of cooked (100 g of raw) Ca-fortified rice could provide one-fifth of the daily recommended intake of Ca (Naila et al., 2019). While Ca fortification as such may not be the most appropriate long-term solution for alleviation of Ca deficiency in developing countries, its benefits certainly emphasize the potential advantages of the enrichment of this major crop with Ca.

Similarly, wheat could be used as a vehicle for improving the Ca intake of consumers. The average consumption of wheat is 67.5 kg per person (FAO/WHO, 2002), with a significant rise in consumption in Asia (especially China and India) (FAOSTAT, 2017). Wheat lines with high concentrations of Ca have been identified. When Ca concentrations were measured in different wheat lines, out of the nine advanced lines of spring bread-making wheat (*Triticum aestivum L*.) from the Portuguese Wheat Breeding Program, the highest concentration of Ca was seen in Nabao and Cultivars 1 and 8, around 480 mg Ca/kg (Gomez-Coronado et al., 2019). Similar concentrations of Ca in bread wheat plants were found by Calderini and Ortiz−Monasterio (2003), 310–516 mg Ca/kg. Ca concentrations for 150-bread wheat genotypes investigated by Pandey et al. (2016) were around 300 mg Ca/kg, with a minimum and a maximum value of 106 and 663.5, respectively. Gomez-Becerra et al. (2010) tested 700 lines of spelled wheat grains and reported Ca concentrations in the range between 132 and 884 mg Ca/kg. In addition, 40% lower concentrations of phytic acid were measured in spelled than in wheat flour (Ruibal-Mendieta et al., 2005). High heritability traits demonstrated for spelled grain show that the new spelled breeding lines with improved concentrations of Ca could be developed (Gomez-Becerra et al., 2010). In the 64-bread wheat and 21-durum wheat varieties commonly grown in Turkey, Ca concentrations varied between 266 and 531, with a mean value of 378 mg Ca/kg in bread wheat, and 215–468 with a mean of 330 mg Ca/kg in durum wheat (Harmankaya et al., 2012).

In summary, biofortification of major staple crops is a widely accepted, low-cost strategy that could provide essential nutrients for people. The challenge is now not only to produce Ca-rich staple crops but to make the Ca bioavailable so as to have a beneficial effect on Ca status of consumers.

## Genetic Diversity and Transgenic Approaches

Genetic marker development (i.e., through the identification of quantitative trait loci (QTL) and underlying genes responsible) is important for studying and manipulating complex traits important to agriculture and for recognizing genetic differences present within the plant species. The approach could greatly accelerate genetic modification of Ca amounts in a number of crops.

Over the years, a number of QTLs for Ca accumulation in grains of certain crops plants (i.e., wheat, rice, sorghum, barley, maize, and pearl millet) have been identified (Peleg et al., 2009; Zhang et al., 2009; Goel et al., 2011; Fedorowicz-Strońska et al., 2017; Sharma et al., 2017). Ca accumulation in hexaploid wheat grains is controlled by multiple loci, located mainly on chromosomes 2A, 3A, 5A, and 6A, one on 5D and one on the 5B chromosome (Alomari et al., 2017).

Nine significant QTLs were associated with Ca concentrations in wheat grains (Peleg et al., 2009). The genome wide association study of Ca concentrations in bread wheat (*Triticum aestivum*) was performed on 353 genotypes from a European wheat diversity panel and demonstrated that the most significant gene for Ca accumulation was located on chromosome 5A (Alomari et al., 2017).

Importantly, the heritability was 0.73 across the 2 years, and, while an environmental effect was present, the relatively moderate heritability would allow for genetic gains to be made in a plant breeding context. Ultimately, both genetic and environmental factors have a significant effect on Ca concentration in wheat and maize grains (Gu et al., 2015; Alomari et al., 2017).

Goel et al. (2011) presented a complete genome wide comparative analysis of the Ca transporter gene family in rice and sorghum. The accumulation of Ca in rice and sorghum involved 31 and 28 transporter genes, respectively; however, not all of these proteins were involved in Ca accumulation *per se* (Goel et al., 2011). In rice, the transporters were distributed on nine out of 12 chromosomes, while sorghum transporters could be found on all except on chromosome 10 (Goel et al., 2011). Phylogenetic analysis divided proteins into four clusters: Ca channel, IIA- and IIB-type Ca, ATP-ases, and a Ca exchanger. What was interesting is that Ca transporters were not evenly distributed but rather clustered on certain chromosomes (Goel et al., 2011). Three QTLs identified in rice plants were mapped in the marker interval STS41t9-RM7 on chromosome 3, RM5271-RM216 on chromosome 10, and RM21-RM3428 on chromosome 11, with 14.3, 18.7, and 13.0% of the variation in Ca accumulation (Du et al., 2013). In rice plants, qCa1-1 loci on chromosome 1 accounted for about 9–14% of phenotypic dissimilarity in Ca concentrations in rice (Garcia-Oliveira et al., 2009). The genotype had a significant effect on Ca concentrations. For example, the lowest concentrations of Ca were measured in the Bacanora T88 wheat line while the concentrations did not differ between the Rayon F89 and a synthetic line (Calderini and Ortiz-Monasterio, 2003), so synthetic hexaploids may be a useful source for increasing Ca concentrations of wheat plants.

The genetic diversity of finger millet has been extensively examined in recent years, and a large collection of germplasm has been investigated with 15 accessions identified as most promising for improving Ca content (Upadhyaya et al., 2011, 2014; Shambhavi et al., 2020). About 82% of the entire genome size of finger millet was assembled, using the modern generation sequencing technologies (Hittalmani et al., 2017). Transport and accumulation of Ca have also been studied across various genotypes, using the high throughput RNA sequencing technology. The low and the high Ca content finger millet grains had different genotypes, GPHCPB1 and GPHCPB45, respectively (Kumar et al., 2015). The highest concentration of Ca was measured in GPHCPB44, GPHCPB45, IE6537, and IE2957 accessions (Yadav et al., 2020). EGEP60 and UGEP78 markers accounted for 6.4 and 13.8% phenotypic variance in finger millet (Yadav et al., 2020).

The exogenous supply of Ca was positively correlated with the expression of Ca sensor genes (Singh et al., 2014). Two Ca-binding proteins were identified in finger millet seeds, calcineurin-B, and calreticulin. Shoots of plants overexpressing calreticulin have higher Ca levels and may be potential targets for genetic manipulation (Broadley and White, 2010).

The mechanism by which Ca is moved and transported into the seed is not completely understood, but some preliminary data suggest that Ca transporters are the main Ca-transporting proteins (Li, 2006). CaM-sensor proteins, localized in the embryo close to the aleurone layer, are controlling the activities of most of the Ca transporters. Higher abundance of CaM around the aleurone layer was seen in the seeds of high grain Ca genotypes, which implies that CaM may be accountable for the high accumulation of Ca in the grains.

Candidate genes that could be applied to enrich Ca concentration have been studied, using Brassica napus genotypes, with the use of an associative transcriptomics approach through an application of the candidate genes selected based on Arabidopsis thaliana-distinguished functions (Alcock et al., 2017).

The leaf Ca concentration was significantly correlated with loci on chromosomes A10, A3, A6, A, C9, C2, and C3. A10 and C9 were the most highly associated loci with Ca content. Flowering locus C (FLC) and suppressor or overexpression of CO1 (SOC1) markers were associated with Ca concentrations in leaves (Alcock et al., 2017). The ACA8 gene was shown as a promising candidate for the control of Ca accumulation in Brassica napus (Alcock et al., 2017). The favorable candidate for influencing the translocation of Ca to particular tissues in crop plants was the gene that encodes nuclear transport factor 2 (NTF2). In Arabidopsis thaliana, five QTLs with 36.4% of the variation in Ca content were recognized (Vreugdenhil et al., 2004). The investigation of the magnitude of variability in a core germplasm collection of pearl millet revealed accessions differing in Ca content from 85 to 249 mg/kg with heritability estimates of over 0.81 (Govindaraj et al., 2020). A strong genetic control was observed for Ca with a lower magnitude of genotype × environment relationships (Govindaraj et al., 2020). Very high heritability traits were days to 50% flowering, 77% for Ca, and the relative performance of accessions for Ca did not differ significantly from one season to another, confirming persistent genetic regulation. Flowering was directly correlated with Ca, so late-flowering accessions had a longer time to collect Ca than early flowering counterparts, and small shrunken grains had higher Ca content (Govindaraj et al., 2020).

Breeding studies demonstrated genotypic variability in common bean varieties in the traits responsible for Ca content. Promising common bean populations with high Ca content have been identified, showing that 80% of the Ca content of common beans is concentrated in the seed coat (Fernandes et al., 2016). A hard-to-cook phenotype in the lpa1 common bean line was associated with redistribution of Ca mainly in the cell walls (Cominelli et al., 2020). However, different results were found in various grain tissues; both higher and lower concentrations of Ca were seen in maize, soybean, and barley between the *lpa* mutants and the wild type (Bryant et al., 2005; Lin et al., 2005; Landoni et al., 2013).

Development of mapping populations, breeding, and mutant lines would be valuable for grouping the varieties according to the grain Ca content. Genome wide association studies that obtain new genetic information could help in identifying the genes involved in Ca accumulation in various grains and in identifying the most appropriate varieties for biofortification. The achievement of Ca biofortification of major crops depends on the extent of characterized germplasm for numerous traits that could be offered to breeders for breeding Ca-rich cultivars.

Transgenic approaches to improve the Ca concentration in plants have also been studied. The Ca content of plants usually increases with a rise in external Ca supply (White, 2001). Ca supply to the plant can be improved by mobilizing soil-available Ca or by improving Ca absorption through a more extensive root system (Puranik et al., 2017). Sequestration of Ca within a plant cell depends on the Ca^2+^/H^+^ antiporter (CAX1) situated on the tonoplast. The manipulation of the CAX gene in Arabidopsis produced twofold more Ca compared with control plants, with no alterations in oxalate levels and growth (Hirschi, 2009; Dayod et al., 2010). Similarly, potatoes, carrots, and lettuce with increased Ca concentration have been produced by an expression of a single copy of the CAX1 gene (Yang et al., 2012). However, the overexpression of the CAX gene promoted Ca deficiency symptoms in vulnerable tissues (Dayod et al., 2010). The Ca concentration of some plants has been increased with overexpression of tonoplast Ca transporters, i.e., in lettuce, watermelon, and potato tubes (Han et al., 2009; Park et al., 2009). Certain Ca sensors and binding genes [i.e., type IIB ATPase, Ca^2+^/H^+^ antiporter (CAX1), calmodulin (CaM), two-pore channel (TPC1), and CaM-dependent protein kinases (CaMK1 and CaMK2)] are proposed as responsible for Ca enhancement and accumulation within the finger millet grains (Figure 2; Puranik et al., 2017).

Cation/proton exchangers (CAX) transporters are good candidates for increasing bioavailable Ca in plants, but the expression should be stimulated cautiously to avoid deficiency symptoms that arise in certain tissues. Important to note, simply increasing the Ca supply to cells is not always providing the expected enhancement of Ca concentration, which clearly means that a leading factor in the process is the Ca transport within the cells.

Modern breeding techniques, i.e., CRISPR/Cas-based tools, offer exciting new opportunities for creating directed genetic diversity. These techniques allow the generation of site-specific genetic diversity and improve the identification of genes underlying certain QTLs (Shan et al., 2013; Yin et al., 2017; Wolter et al., 2019). Furthermore, the procedures enable manipulation of wild relatives of certain crops as a beneficial source of allele mining, open up the genetic diversity from uncultured species, and accelerate the domestication process significantly (Shan et al., 2013; Kumlehn et al., 2018). In addition, CRIPS enables immediate generation of genomic diversity and fine-tuning of desirable traits (Wolter et al., 2019). Finally, novel plant breeding methods could speed up the production of crop plants with desired traits that will meet the challenges related to the food supply, climate change, and environmental sustainability (Ahmar et al., 2020).

In summary, the genetic diversity of Ca content in various crop varieties has been examined. Certain markers and genome sequences that control Ca content of major crops are already identified, but they remain to be adequately scrutinized. The identification of all the genes that control the accumulation of Ca into various grains requires an extensive genome-wide examination. Identification of these genes can help in the development of transgenic plants to further aid in understanding Ca transport in plants. Modern plant-breeding approaches should be employed for faster identification and implementation of exact molecular mechanisms for crop improvement.

## Conclusion and Recommendations for Further Research

Calcium is an essential plant nutrient vital for human health. Dietary Ca deficiency remains prevalent among people in developing and developed countries, causing a number of diseases: osteoporosis, inadequate bone mineralization, hypertension, diabetes, and various forms of cancers. At the same time, the demand for staple food crops is increasing and is expected to get higher. More nutrient dense staple food crops could provide a solution to the Ca malnutrition-related problems. Food fortification and supplementation have not been successful long-term strategies for resolving the inadequacy of intake of many other nutrients, so they probably will not work for Ca either.

On the other hand, the biofortification of main crops could be an effective strategy for increasing Ca content of crop plants. Breeding staple crops with higher Ca concentrations is a sustainable long-term strategy for alleviating ensuing Ca deficiency by providing sufficient amounts of Ca for populations dependent on plant foods as a basic food source and those with limited and/or restricted access to dairy foods.

Before a Ca biofortification program moves forward, it is important to identify physiological and evolutionary restraints to improving Ca concentration of edible portions of major crops to define the most suitable crop plants and crop parts for planned interventions. Breeding for increased Ca bioavailability is a perplexing task as bioavailability depends on a number of factors: the genotype, the accumulation of antinutrients, the mineral interactions, the colonization of plants by fungi, the Ca uptake systems, etc. Similarly, a rapid screening method of Ca bioavailability could be developed.

A better understanding of the Ca physiology of plants is needed to design crop breeding strategies that will increase both the Ca concentration of grains and also the yield.

Finger millet, a crop with a higher grain Ca concentration, is a great candidate for learning about mechanisms that contribute to Ca accumulation in grain crops and should be used as an example for increasing the Ca content of other most frequently consumed staple crops. Certain QTLs for Ca accumulation and augmented Ca uptake are already known, but more work is needed in order to completely elucidate loci involved in target crops. Breeding programs for the development of plant crops with the ability to accumulate more Ca from the soil and translocate it to the edible parts should be pursued. The identification of food preparation methods that could reduce the negative impact of antinutrients, oxalates and phytates, on Ca bioavailability is essential.

Calcium-rich grains have the potential to improve the Ca status of consumers, especially those with cereal-based dietary patterns and those with limited intake of milk and dairy products. The development of Ca-biofortified cereals requires well-developed methodologies for the evaluation of Ca bioaccessibility and appropriate strategies for assessing the efficacy of Ca-biofortified crops in improving the Ca status of consumers. Both *in vitro*, i.e., caco-2 cells, and *in vivo* methods should be developed and employed in order to adequately assess the bioaccessibility of Ca of projected Ca biofortified cereal grains. Optimal processing and food preparation practices are the key elements in this process. Procedures, such as fermentation, soaking, processing, and cooking, are all well-known for their inhibitory effect on phytic acid activity in cereals grains. The antinutritional effects of phytate are significantly reduced by these procedures, which lead to improved Ca bioaccessibility. Potential long-term negative effects of reduced phytate levels on human health should be taken into account. Environmental conditions that affect the Ca content of crops should be carefully examined and adequately addressed where possible.

Further research is needed to determine the key drivers of Ca concentrations in various crops, the link between the soil and crop characteristics, and, finally, the effect that climate and agricultural practices may have on Ca accumulation in an assortment of crop plants. Policies that promote and encourage the development and application of Ca-biofortified cereals in developed countries should be part of the general approach to improving dietary intake of Ca in most vulnerable populations. Finally, in order for Ca-biofortified cereals to be successfully developed, tested, and, finally, adequately implemented in susceptible population groups, research, political will, partnership, investment, collaboration, government engagement, leadership, and constant innovation of applicable methods are needed. A multidisciplinary approach and collaboration among health, food, agriculture, and social protection systems are of crucial importance in this instance.

## Author Contributions

MK and JS conceptualized the manuscript. MK wrote the manuscript and prepared the manuscript for submission. JS contributed critically in revising the draft and updating the manuscript for publication. Both authors contributed to the article and approved the submitted version.

## Conflict of Interest

The authors declare that the research was conducted in the absence of any commercial or financial relationships that could be construed as a potential conflict of interest.
